# Development and validation of a nomogram to predict the recurrence of eyelid sebaceous gland carcinoma

**DOI:** 10.1002/cam4.6126

**Published:** 2023-06-30

**Authors:** Zihan Nie, Jialu Geng, Xiaolin Xu, Ruiheng Zhang, Dongmei Li

**Affiliations:** ^1^ Beijing Ophthalmology & Visual Science Key Laboratory Beijing Tongren Eye Center, Beijing Tongren Hospital, Capital Medical University Beijing China; ^2^ Beijing Tongren Eye Center, Beijing Key Laboratory of Intraocular Tumor Diagnosis and Treatment, Beijing Ophthalmology&Visual Sciences Key Lab, Medical Artificial Intelligence Research and Verification Key Laboratory of the Ministry of Industry and Information Technology Beijing Tongren Hospital, Capital Medical University Beijing China; ^3^ Dong Jiao Min Lane Beijing China

**Keywords:** eyelid sebaceous gland carcinoma, nomogram, prediction, recurrence, survival

## Abstract

**Purpose:**

Eyelid sebaceous gland carcinoma (SGC) is a malignancy with fatal risk, high recurrence rate, and pagetoid spread. Thus, recurrence risk prediction and prompt treatment are extremely important. This study aimed to develop a nomogram to predict SGC recurrence based on potential risk factors.

**Methods:**

We conducted a retrospective study to train and test a nomogram based on the clinical data of 391 patients across our hospital (304) and other grass‐roots hospitals (87). After Cox regression, predictors included in the nomogram were selected, and sensitivity, specificity, concordance index (C‐index), etc., were calculated to test their discrimination ability.

**Results:**

After a median follow‐up period of 4.12 years, SGC recurred in 52 (17.11%) patients. The 1‐, 2‐, and 5‐year recurrence‐free survival rates were 88.3%, 85.4%, and 81.6%, respectively. We examined five risk factors, such as lymph node metastasis at initial diagnosis (hazard ratio [HR], 2.260; 95% confidence interval [CI], 1.021–5.007), Ki67 (HR, 1.036; 95% CI, 1.020–1.052), histology differentiation degree (HR, 2.274; 95% CI, 1.063–4.865), conjunctival pagetoid infiltration (HR, 2.100; 95% CI, 1.0058–4.167), and orbital involvement (HR, 4.764; 95% CI, 1.436–15.803). The model had good discrimination in both internal and external test sets. The model had good discrimination in both internal and external test sets. The sensitivity of the internal test and external test set were 0.722 and 0.806, respectively, and specificity of the internal test and external test set were 0.886 and 0.893, respectively.

**Conclusion:**

We examined the potential risk factors for eyelid SGC recurrence and constructed a nomogram, which complements the TNM system in terms of prediction, indicating that our nomogram has the potential to reach clinical significance. This nomogram has the potential to assist healthcare practitioners in promptly detecting patients who are at an elevated risk and in tailoring clinical interventions to meet their individualized needs.

## 1 INTRODUCTION

Eyelid sebaceous gland carcinoma (SGC), commonly arising from the epithelium of the meibomian and Zeis glands, is a cutaneous malignant neoplasm occurring in the periocular area.^1^ The worldwide incidence of eyelid cancer is approximately 5.1–15.7 cases/100,000 individuals annually.^2^ In China, SGC is considered the second most common malignant tumor of the ocular adnexa and eyelid, following basal cell carcinoma, accounting for approximately 34.24%–38.6%.^3^, ^4^, ^5^ Eyelid SGC usually presents as diffuse eyelid thickening at the earlier stage and isolated masses at the later stage, so it is easily confused with blepharoconjunctivitis or chalazion. Therefore, SGC can easily be missed or misdiagnosed, thereby delaying treatment.^6^

SGC tends to recur postoperatively due to its multicentric origin and pagetoid spread.^6^ The overall local recurrence rate of SGC ranges from 5% to 18%.^7^, ^8^, ^9^, ^10^ Poor prognostic risk factors for SGC include the tumor's largest diameter at >20 mm,^10^ worse T category,^7^, ^9^, ^10^, ^11^ pagetoid spread,^6^, ^12^ and orbital tumor extension.^11^ Therefore, recurrence risk factors of SGC should be examined based on clinical data, and a nomogram prediction model should be constructed for personalized prediction to prevent recurrence and improve patient management.

The American Joint Committee on Cancer (AJCC) guidelines for the classification of eyelid carcinoma include the tumor's largest diameter, the extent of tumor invasion to the periphery (tarsal plate, eyelid margin, ocular or intraorbital structure, or periorbital tissues), and lymph node and distant metastases. However, the pathological differentiation degree, Ki67, and other factors can also affect patient prognosis.^13^, ^14^ Therefore, more potential risk factors can be incorporated into the nomogram risk scoring system to predict patients' recurrence‐free survival rate.

## 2 METHOD

### 2.1 Patients

This study was a large retrospective study exploring recurrence prediction factors. The Ethics Committee of Beijing Tongren Hospital, Capital Medical University (No. TRECKY2018‐056) approved this study. Additionally, the study was conducted following the Declaration of Helsinki. Informed consent was signed after the purpose of the study was thoroughly explained to the patients and their family members.

**FIGURE 1:**
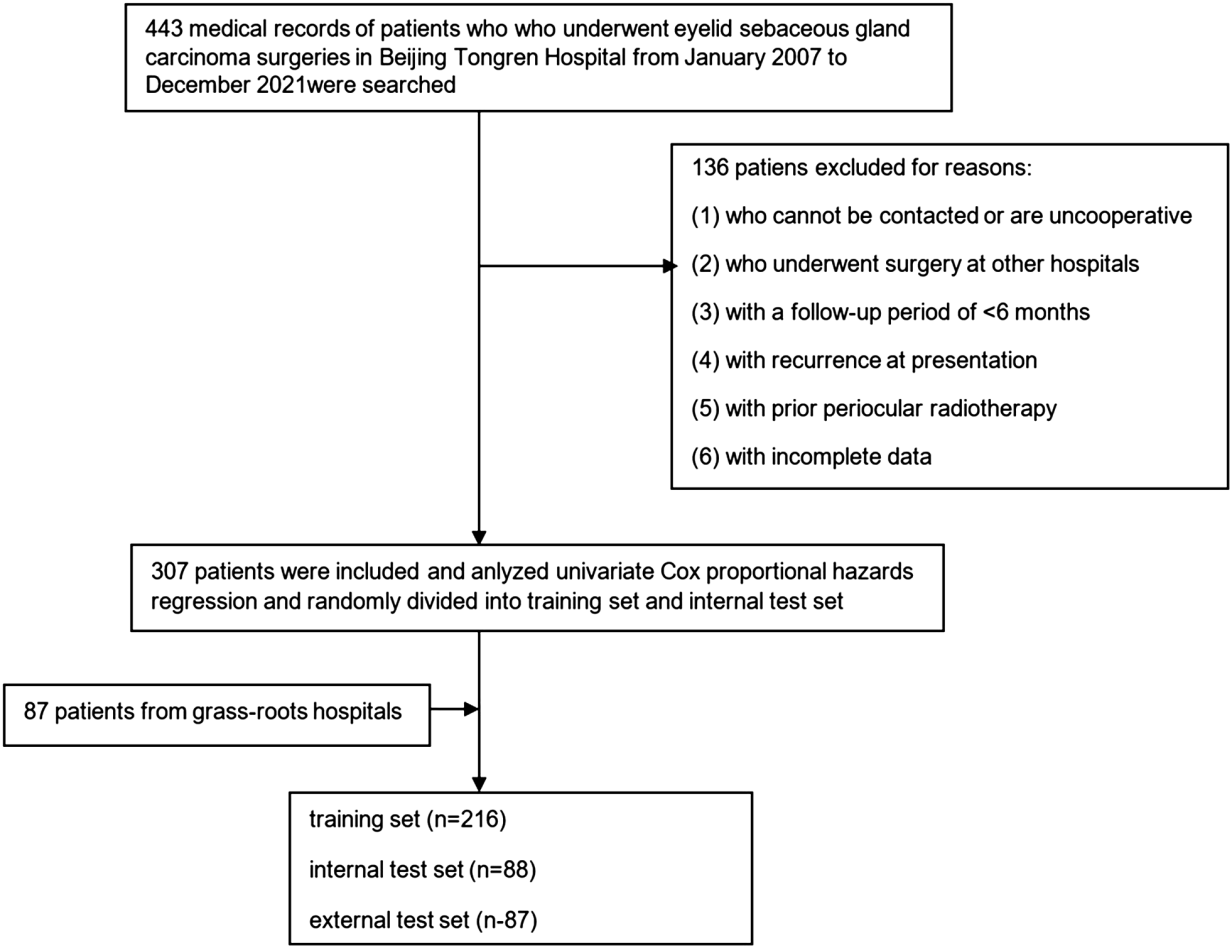
Flowchart of the nomogram.

The study collected the medical records and pathological data of patients who underwent SGC surgeries in Beijing Tongren Hospital from January 2007 to December 2021. The inclusion criteria were (1) patients diagnosed with eyelid SGC by our hospital's outpatient and pathology departments, (2) patients who were surgically treated and pathologically examined, and (3) complete medical record and pathology data. The exclusion criteria were patients (1) who cannot be contacted or are uncooperative, (2) who underwent surgery at other hospitals, (3) with a follow‐up period of <6 months, (4) with recurrence at presentation, (5) with prior periocular radiotherapy, and (6) with incomplete data. After screening, we included 304 patients in the study. The flowchart is illustrated in Figure 1.

**FIGURE 2:**
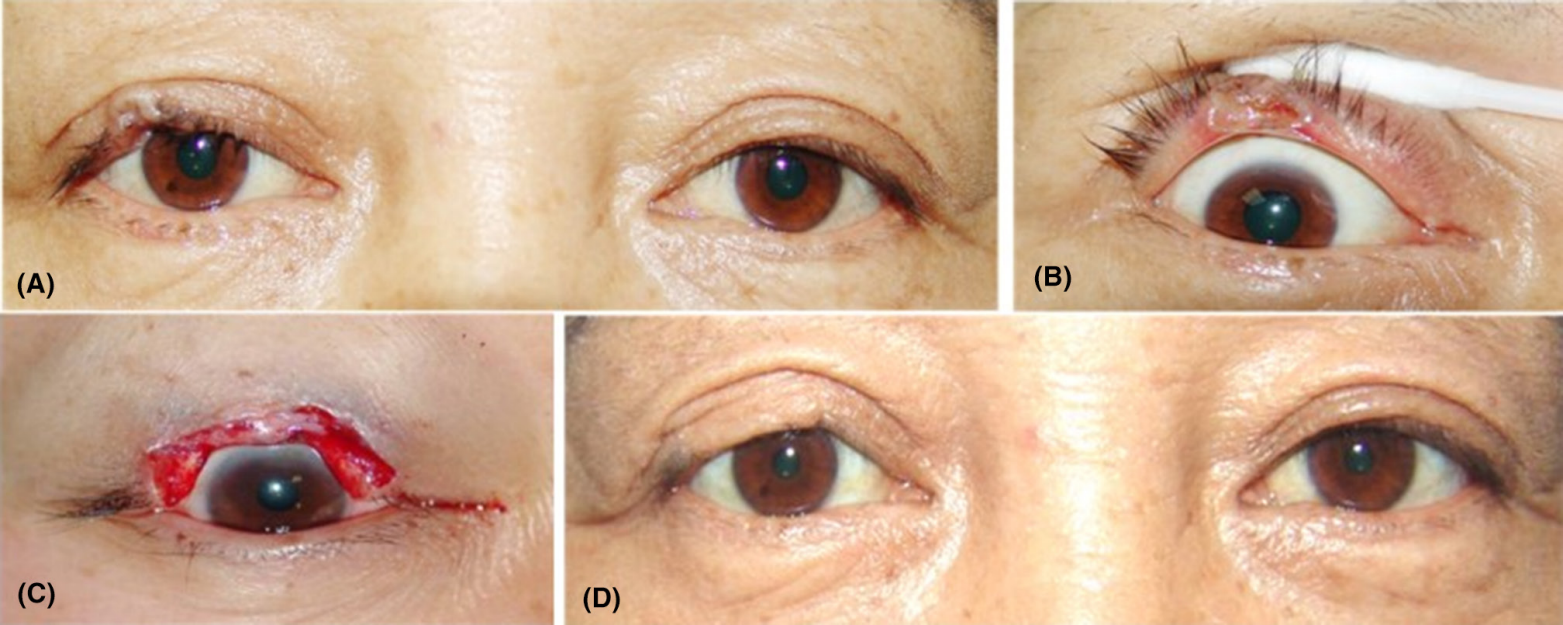
Right upper lid tumor (T1). (A) Ocular surface of the tumor; (B) conjunctival surface of the tumor; (C) expanded mass excision; (D) postoperative result.

All patients were treated with margin control with a frozen section during the surgical procedures. Subsequently, the resected tumor margins were marked, and frozen sections were immediately processed for histopathological examination. All the resected tissue sections from the outer edge to the inner side underwent pathological examination to ensure the absence of tumor cells. If any tumor cell was detected, wide mass excision was performed until the resection margin tissue had no tumor cells after microscope examination of the frozen sections. Finally, the tumor was excised using a >3‐mm margin of clinically uninvolved tissue (safe margin). Afterward, the eyelid was reconstructed based on the extent of the eyelid defect. Individualized reconstruction was performed based on the patient's specific condition (Figure 2). Enucleation or orbital exenteration was performed for tumors with adjacent periocular or periorbital tissue invasion. Eventually, lymphadenectomy was performed for the confirmed presence of nodal metastasis, and radiotherapy was used as adjuvant therapy.

Each patient was followed up regularly to monitor their condition postoperatively (each patient had at least two postoperative follow‐up records), and main indicators, such as tumor recurrence and metastasis, were checked during the follow‐up. The primary outcomes were recurrence and the time from surgery to the onset of the first recurrence event. Specialized physicians diagnosed tumor recurrence through pathological and imaging examinations.

### 2.2 Data collection

Based on the Health Information System and Electronic Medical Records, clinical data, such as age, sex, initial tumor location, tumor size (including length, width, and depth), tumor category based on the 8th edition of AJCC staging system for eyelid SGC,^15^ recurrence and metastatic situation, lymph node metastasis at initial diagnosis, local involvement (e.g., presence of orbital involvement or conjunctival pagetoid invasion), tumor pathological differentiated degree, and positive Ki67 tumor marker expression. Relevant information from the 87 patients from grass‐roots hospitals was also collected as an external test set to further test the extrapolation capability of our nomogram prediction model.

The tumor tissue specimens were formaldehyde‐fixed, alcohol‐dehydrated, and paraffin‐embedded. Subsequently, after deparaffinization, the specimens were cut into 3–5‐μm thickness for hematoxylin–eosin staining and Ki67 antibody immunostaining. The pathologic differentiation degree of SGC can be classified as well, moderately, and poorly differentiated.^16^ Well‐differentiated SGC is characterized by foamy sebocyte‐like cells and lobules with sebaceous differentiation. Moreover, poorly differentiated SGC is presented as pleomorphic cells with prominent nucleoli, pleomorphic nuclei, and amphophilic‐positive cytoplasm.^15^

### 2.3 Statistical analysis

R software (R version 4.2.2, Foundation for Statistical Computing) and SPSS software (version 25.0, Foundation for Statistical Computing, IBM) were used for analysis. The “rms,” “foreign,” and “survival” packages in R were used to develop a nomogram. Frequency (percentage) was described as categorical variables, and continuous variables with normal and skewed distributions were presented as mean ± standard deviation and median (interquartile range), respectively. The Kaplan–Meier (K‐M) method was used to calculate the survival and recurrence rates, and the log‐rank test was used for comparison. A *p*‐value of <0.05 was considered statistically significant.

To select the predictors included in the nomogram, univariate Cox proportional hazards regression was used initially to screen all potential recurrence predictors for SGC. Subsequently, potential statistically significant factors (*p* < 0.1 in univariate Cox regression) were included in the multivariate Cox regression. The forward stepwise method (likelihood ratio) was used in this step. The hazard ratios (HRs) with a 95% confidence interval (CI) in the Cox regression analysis were recorded (Table 3). Finally, a nomogram was established based on the multivariate Cox proportional hazard regressions to calculate the prognostic index (PI) and predict recurrence‐free survival probability at 1, 2, and 5 years after treatment.

We randomly divided 304 individuals into the training (216, 70%) and internal test sets (88, 30%) using the “caret” package in R. To evaluate the discriminatory ability of the nomogram prediction model, the concordance index (C‐index), sensitivity, specificity, and area under the curve (AUC) of the time‐dependent receiver operating characteristic curve was calculated.

Each risk factor was calculated, and their scores were added to obtain the total points equal to the PI. The patients were divided into four risk categories based on the PI score. Those with a PI score of <60, 60–80, 80–100, and >100 were classified as low, medium, high, and very high risk, respectively. K‐M analysis and log‐rank test were used to compare the differences in recurrence between the different risk subgroups. Using the T category as the only predictor, C‐index, sensitivity, specificity, and AUC were also calculated to compare the discrimination accuracy of the TNM staging system. Additionally, the net reclassification improvement (NRI) was calculated to indicate the advantages of the nomogram over the T category. Moreover, the bootstrapping method achieved relatively unbiased performance estimates (500 repetitions). All analyses were two‐sided, and *p* < 0.05 was considered statistically significant. The calibration curves of the nomogram for 1‐, 2‐, and 5‐year recurrence‐free survival rates were plotted to assess the agreement between the predicted and actual outcomes.

## 3 RESULTS

### 3.1 Clinical characteristics of the patients

We included 304 eyes of 304 patients from Beijing Tongren Hospital, comprising 129 (42.43%) and 175 (57.57%) men and women, respectively. The mean age of the patients at initial diagnosis was 63.00 ± 12.51 years (range, 29–90 years). Table 1 shows the demographic and clinical characteristics of the patients. Most tumors were found in the upper eyelid (179, 58.89%), followed by lower eyelids (99, 32.57%) and elsewhere (both the upper and lower eyelids, 1.64%, inner and outer canthal, 3.29% and 3.62%, respectively). Notably, 11, 15, and 24 patients had lymph node metastasis on their initial visit, orbital involvement, and conjunctival pagetoid infiltration, respectively.

**TABLE 1:** Basic characteristics and clinical findings of the participants.

Variables	*n* (%)/Mean ± SD/median (IQR)
Total number of patients	304
Age	63.00 ± 12.51
Range	29–90
Gender	
Male	129 (42.43%)
Female	175 (57.57%)
Affected eye	
Right	142 (46.71%)
Left	162 (53.29%)
Location	
Upper eyelid	179 (58.89%)
Lower eyelid	99 (32.57%)
Both eyelids	5 (1.64%)
Inner canthus	10 (3.29%)
Lateral canthus	11 (3.62%)
Tumor T stage	
T1	147 (48.36%)
T2	103 (33.88%)
T3	32 (10.53%)
T4	22 (7.24%)
Largest tumor basal diameter (mm)	10 (11)
Time to presentation (months)	6 (14.5)
Range	0.25–120
Lymph node metastasis at first diagnosis	3 (0.99%)
Orbital involvement	15 (4.93%)
Conjunctival pagetoid infiltration	24 (7.77%)
Ki 67 (%)	40 (45)
Histology differentiation	
Well or moderately differentiated	239 (78.62%)
Poorly differentiated	65 (21.38%)

*Note*: T stage, tumor category according to the 8th edition of the American Joint Committee on Cancer (AJCC) staging system.

Abbreviations: IQR, interquartile range; SD, standard deviation.

After a median follow‐up period of 4.12 years (range, 1.20–6.50 years), 52 (17.11%) patients had SGC recurrence. Based on TNM staging, 18, 22, 4, and 8 patients were diagnosed as stage T1, T2, T3, and T4, respectively, at initial diagnosis. Additionally, 18 (5.92%) patients developed metastasis postoperatively. The metastatic sites included nasal lymph nodes (2, 0.66%), cervical lymph nodes (4, 1.32%), parotid glands (9, 2.96%), maxillofacial lymph nodes (2, 0.66%), and preauricular lymph nodes (1, 0.33%). Moreover, two (0.66%) patients had systemic metastasis, and another two patients presented with lymph node metastases at two sites. The median recurrence time was 35 months (range, 0–154). Table 2 shows the detailed follow‐up information. Based on the K‐M method, the 1‐, 2‐, and 5‐year recurrence‐free survival rates were 88.3%, 85.4%, and 81.6%, respectively.

**TABLE 2:** Follow‐up results of SGC patients.

Variables	*n* (%)/Mean ± SD/median (IQR)
Follow‐up time median (years)	4.12 (1.20–6.50)
Patients with metastasis	18 (5.92%)
Nasal lymph nodes	2 (0.66%)
Cervical lymph nodes	4 (1.32%)
Parotid glands	9 (2.96%)
Maxillofacial lymph nodes	2 (0.66%)
Preauricular lymph node metastasis	1 (0.33%)
Systemic metastasis	2 (0.66%)
Lymph node metastasis at more than two sites	2 (0.66%)
Total mortality number	3 (0.99%)
Patients with a second primary tumor	3 (0.99%)
Breast cancer	1 (0.33%)
Lung cancer	1 (0.33%)
Colon cancer	1 (0.33%)
Patients with recurrence	52 (17.11%)
T1	18 (34.62%)
T2	22 (42.30%)
T3	4 (7.69%)
T4	8 (15.38%)

*Note*: T stage, tumor category according to the 8th edition of the American Joint Committee on Cancer (AJCC) staging system.

Abbreviation: IQR, interquartile range.

### 3.2 Results of uni‐ and multivariable analyses

Before developing the nomogram, predictors were screened using uni‐ and multivariable analyses (Table 3). The univariate analysis estimated that the potential risk factors for recurrence were lymph node metastasis at initial diagnosis (HR, 2.260; 95% CI, 1.021–5.007), Ki67 (HR, 1.036; 95% CI, 1.020–1.052), histology differentiation degree (HR, 2.274; 95% CI, 1.063–4.865), conjunctival pagetoid infiltration (HR, 2.100; 95% CI, 1.058–4.167), and orbital involvement (HR, 4.764; 95% CI, 1.436–15.803). The multivariate Cox regression analysis was performed using the forward stepwise method (likelihood ratio), and the results showed that the five potential predictors were all statistically significant, and they were eventually included in the nomogram.

**TABLE 3:** Uni‐ and multivariable Cox proportional hazards regression analyses for the predictors of recurrence.

	Univariable		Multivariable (full‐model)	
	HR (95% CI)	*p*	HR (95% CI)	*p*
Age (years)	0.994 (0.974–1.015)	0.600		
Sex	0.931 (0.538–1.609)	0.797		
Classification of T stage		0.551		
T2 versus T1	1.206 (0.671–2.168)	0.532		
T3 versus T1	0.503 (0.151–1.671)	0.262		
T4 versus T1	1.130 (0.392–3.258)	0.821		
Larges tumor basal diameter (mm)	1.017 (0.988–1.047)	0.249		
Location of tumor		0.517		
Location 2 versus Location 1	0.000 (0.000–3.913E+225)	0.968		
Location 3 versus Location 2	1.720 (0.681–4.343)	0.251		
Course of disease (months)	1.001 (0.988–1.014)	0.884		
Lymph node metastasis at first diagnosis	13.877 (6.830–28.195)	<0.01*	2.260 (1.021–5.007)	0.044*
Ki 67 (%)	1.056 (1.042–1.071)	<0.01*	1.036 (1.020–1.052)	<0.001*
Histology differentiation (well, moderately, or poorly differentiated)	7.878 (4.486–13.832)	<0.01*	2.274 (1.063–4.865)	0.034*
Orbital involvement	2.840 (0.883–9.135)	0.080	4.764 (1.436–15.803)	0.011*
Conjunctival pagetoid infiltration	8.532 (4.842–15.034)	<0.01*	2.100 (1.058–4.167)	0.034*

*Note*: T stage: tumor category according to the 8th edition of the American Joint Committee on Cancer (AJCC) staging system; Location 1: upper eyelid or lower eyelid; Location 2: both eyelids; Location 3: inner canthus or lateral canthus.

Abbreviations: CI, confidence interval; HR, hazard ratio; SE, standard error.

* Statistically significant.

### 3.3 Prediction efficiency of the nomogram

**FIGURE 3:**
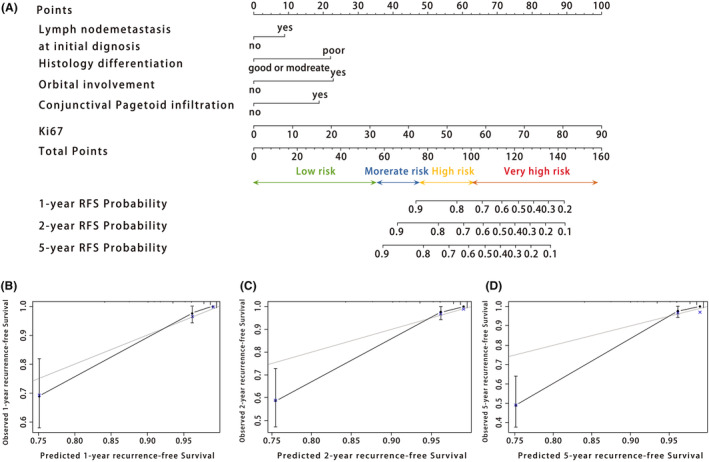
Nomogram for recurrence‐free survival (RFS). (A) Nomogram to predict the probability of RFS at 1, 2, and 5 years. Calibration plots for RFS probability at 1 (B), 2 (C), and 5 years (D). The vertical lines represent the 95% confidence intervals of the estimates. The gray lines represent the ideal lines. Black dot: predicted probabilities based on the nomogram; blue cross: bootstrap‐corrected estimates. B = 500 repetitions for bootstrapping. RFS, recurrence‐free survival.

A prognostic nomogram, including the independent predictors of SGC recurrence, was constructed with the principle that a patient's total risk score is calculated from the sum of the scores for each risk factor (Figure 3A).

The predictive discrimination ability of the training set had an AUC, sensitivity, specificity, and C‐index of 0.963 (95% CI, 0.928–0.997), 0.941, 0.890, and 0.920, respectively. Meanwhile, the AUC, sensitivity, specificity, and C‐index of the internal test set were 0.851 (95% CI, 0.731–0.917), 0.722, 0.886, and 0.817, respectively, and those of the external test set were 0.891 (95% CI, 0.819–0.964), 0.806, 0.893, and 0.807, respectively. The ROC (receiver operating characteristic) curves of train set, internal test set and external test set were shown in the Appendix S1. However, those of the TNM system were 0.500 (95% CI, 0.416–0.584), 0.538, 0.488, and 0.494, respectively. Table 4 shows the NRI between the nomogram and T category at 1, 2, and 5 years. The calibration plots were constructed to exhibit the differences between the predicted and actual values. The recurrence‐free survival probability at 1, 2, and 5 years was generally consistent with observations (Figure 3B–D). The calibration curves of the nomogram for 1‐, 2‐, and 5‐year recurrence‐free survival rates in internal test set and external test set were detailed in the Appendix S1.

**TABLE 4:** Predictive discrimination ability of the nomogram and TNM staging system.

	Nomogram			TNM
	Train set	Internal test set	External test set	
Number	216	88	87	304
Sensitivity	0.941	0.722	0.806	0.538
Specificity	0.890	0.886	0.893	0.488
C‐index	0.920	0.817	0.807	0.494
AUC (95% CI)	0.963 (0.928–0.997)	0.851 (0.731–0.971)	0.891 (0.819–0.964)	0.500 (0.416–0.584)
NRI (1‐year) (95% CI)	1.158 (0.734–1.635)	1.166 (0.135–1.649)	1.189 (0.779–1.589)	—
NRI (2‐year) (95% CI)	1.296 (1.004–1.704)	0.965 (0.455–1.573)	1.145 (0.771–1.541)	—
NRI (5‐year) (95% CI)	1.438 (1.1335–1.750)	1.231 (0.813–1.739)	1.167 (0.879–1.663)	—

Abbreviations: CI, confidence interval; C‐index, concordance index; NRI, net reclassification improvement.

**FIGURE 4:**
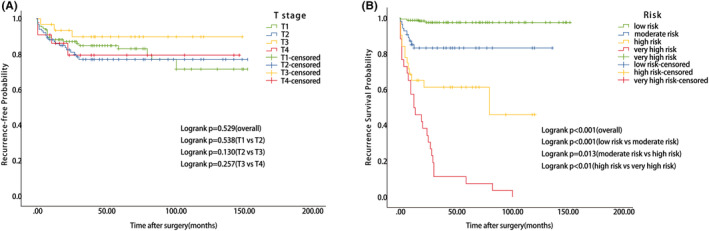
The discrimination ability of the nomogram (B) and T category. (A) Kaplan–Meier curves of RFS based on the T category. (B) Kaplan–Meier curves of RFS for the low, moderate, high, and very high risk.

Furthermore, the recurrence risk of SGC was graded based on the patients' PI score. The K‐M curves were generated based on the T category of the TNM staging system and risk subgroups. The K‐M curves based on the T category did not exhibit satisfying discrimination ability because of the obvious overlap of the survival curves [overall *p* = 0.529; *p* = 0.538 (T1 vs. T2); *p* = 0.130 (T2 vs. T3); *p* = 0.527 (T3 vs. T4)] (Figure 4B). However, the degree of discrimination was evident when K‐M curves were plotted based on the risk category [overall *p* < 0.01; *p* < 0.01 (low vs. moderate risk); *p* = 0.013 (moderate vs. high risk); *p* < 0.01 (high vs. very high risk)] (Figure 4A).

## 4 DISCUSSION

This study aimed to conduct long‐term follow‐up and analyze the prognosis of patients with eyelid SGC, providing detailed patients' clinical characteristics and recurrence information. In this study, the recurrence, nodal metastasis, and mortality rates of SGC were 11.7%, 5.59%, and 0.99%, respectively. However, our study's recurrence rate was lower than that of Shields et al.^7^ in the United States (11/60.18%), higher than that of Choi et al.^8^ in Korea (2/40.5%), and similar to that of Ford et al.^9^ in the United States (7/65.11%). Thus, the recurrence rate of SGC in the Chinese population is generally consistent with that of populations in other countries. The discrepancies may be mainly due to differences in the sample size, surgical technique, ethnicity, and environment.

We constructed nomograms based on five independent risk factors, such as lymph node metastasis at initial diagnosis, Ki67, histology differentiation (well‐, moderately, or poorly differentiated), orbital involvement, and conjunctival pagetoid infiltration. In our investigation, patients who presented with lymph node metastasis at the time of their initial diagnosis displayed a 90.9% recurrence rate (10/11), with two of them subsequently developing over two lymph node metastases. Ki67 is a potent marker for cellular proliferation, and its primary function involves acting as a biosurfactant that promotes the dispersion of chromosomes during mitosis.^17^ Previous studies showed that Ki67 is a prognostic marker in many tumors, such as colorectal cancer, breast cancer, lymphoma, prostate cancer, and laryngeal cancer.^18^, ^19^, ^20^, ^21^, ^22^ Additionally, the Ki67 labeling index (LI) of SGC (46.1% ± 3.0%) was higher than that of normal conjunctiva (6.8% ± 32.3%).^23^ Similarly, high Ki67 expression was associated with poor outcomes in eyelid SGC recurrence and lymph node metastasis.^24^ Well‐differentiated cells of SGC are usually characterized by foamy sebocyte‐like cells with cytoplasm vacuolization. By contrast, many mitotic figures represent poor histologic differentiation in basophilic pleomorphic cells with prominent nucleoli.^25^ These poorly differentiated cells show metabolically active growth with intense aggressiveness and are poor prognostic markers. In this study, the recurrence rate of patients with orbital invasion was 42.6% (3/7). Although two patients underwent secondary surgery for recurrence, they still died because of systemic metastasis or parotid lymph node metastasis. SGC with pagetoid infiltration of the conjunctival epithelium is a more infiltrative carcinoma because it tends to skip from the original tumor to other areas,^26^ indicating its potential to increase recurrence risk.

Esmaeli et al.^6^ have established the correlation between the T category of the 7th edition of the AJCC TNM staging system and patient outcomes in sebaceous carcinoma of the eyelid in previous studies. They found that tumors classified as T3a or worse were linked with a higher incidence of metastasis and tumor‐related mortality, but no significant correlation was found with local recurrence. Conversely, a Japanese study conducted by Watanabe et al.^27^ reported a significant association between T category and tumor recurrence (*p* = 0.01). Kaliki et al.^11^ reported that the metastasis rate and death due to metastasis were proportional to the tumor category, but the correlation between recurrence and T staging was not discussed. In 2019, Hsia et al.^28^ observed that the Kaplan–Meier curve based on the 8th edition of T category crossed, indicating that poor prognostic outcomes were not strongly associated with the severity of the stages.

A nomogram is a graphical device that enables the visualization of a patient's prognostic risk and facilitates the creation of customized management strategies based on individual patient characteristics.^29^ Notably, nomograms are renowned for their superior accuracy, specificity, and sensitivity. Reliable nomograms can be of immense value in predicting the prognosis of eyelid tumors and other malignancies, assisting patients in decision‐making, and aiding healthcare providers in personalizing clinical treatments.^30^, ^31^, ^32^, ^33^ The traditional AJCC classification system focuses on the tumor's largest diameter and invasion profile. However, our retrospective study showed that the degree of pathological differentiation, Ki67 expression, and pagetoid infiltration of the conjunctival epithelium could also increase the risk of recurrence, with specific predictive value. Compared with T staging, the nomogram prediction model showed higher sensitivity and specificity in both internal and external test sets, demonstrating reasonable extrapolation. Furthermore, by contrasting the K‐M curves, the nomogram‐based risk staging system showed much better discrimination compared with the AJCC system. Thus, the developed nomogram significantly improved the accuracy of predicting the recurrence rate of SGC. Therefore, the nomogram will be a useful tool in guiding physician decision‐making. For example, more frequent follow‐ups could be conducted in patients with higher recurrence risk.

In previous research, Zhou et al.^34^ and Gu et al.^35^ investigated the risk factors and developed nomograms for overall survival and metastasis in patients with SGC, respectively. These studies have shown that nomograms can aid clinicians in making decisions regarding disease management and treatment. The present study is the first to utilize a nomogram for predicting the recurrence of eyelid SGC; thus, enhancing the prognostic prediction models for SGC. However, this study has some limitations. First, due to fewer follow‐up data at year 3, our calculated recurrence‐free survival predictions focused on 1, 2, and 5 years after treatment. Second, despite being a comprehensive retrospective study of eyelid SGC, our sample size remains restricted. Additionally, all participants were of Chinese descent, and as such, the findings may not be applicable to other ethnic populations. Third, patients were recruited from a single tertiary hospital in a particular city, and our validation set was limited to a few primary hospitals, thereby lacking a large external validation set. In addition, potential predictors may not be limited to the five predictors included in the study, and other predictors, such as radiotherapy and perineural invasion, were not considered.

In conclusion, statistically significant risk factors were identified for eyelid SGC recurrence, and a well‐discriminated nomogram was constructed based on these factors. The nomogram enables individualized patient risk prediction and facilitates patient management. Thus, the present prediction model can be potentially applied in clinical practice.

## AUTHOR CONTRIBUTIONS

**Zihan Nie:** Data curation (lead); investigation (equal); writing – original draft (equal); writing – review and editing (equal). **Jialu Geng:** Formal analysis (equal); methodology (equal); software (lead); validation (lead); visualization (lead); writing – original draft (lead). **Xiaolin Xu:** Investigation (equal); methodology (equal); supervision (equal). **Ruiheng Zhang:** Software (equal); writing – review and editing (lead). **Dongmei Li:** Conceptualization (supporting); supervision (lead).

## FUNDING INFORMATION

This work was supported by the National Natural Science Foundation of China(grant number 82071005).

## CONFLICT OF INTEREST STATEMENT

All authors declare that the research was conducted without any commercial or financial relationships that could be construed as a potential conflict of interest.

## ETHICS APPROVAL

This study involves human participants and was approved by The Ethics Committee of Beijing Tongren Hospital, Capital Medical University (No. TRECKY2018‐056). Participants gave informed consent to participate in the study before taking part.

## Supporting information


Appendix S1

## Data Availability

The datasets generated or analyzed during this study are available from the corresponding author upon reasonable request.
