# Supplementary material for: Development and validation of a nomogram to predict the recurrence of eyelid sebaceous gland carcinoma
Source: Cancer Med. 2023 Jun 30;12(14):14912–21. doi: 10.1002/cam4.6126 (PMC10417194; doi:10.1002/cam4.6126)
Supplement: Supplementary file 1 — Appendix S1 [file CAM4-12-14912-s001.pdf]

Appendix

Fig 1 ROC(receiver operating characyeristic) curves of train set

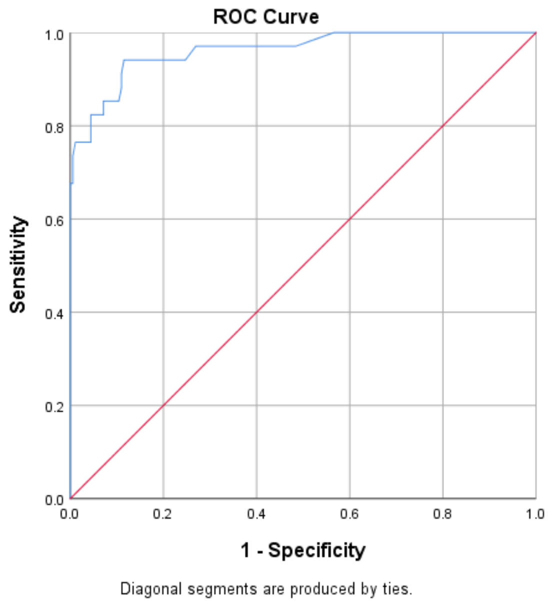

a.

Fig2. Fitted ROC curve of PI index with "KM" method in 1 year (a),2 years(b) and 5 years(c) of train set

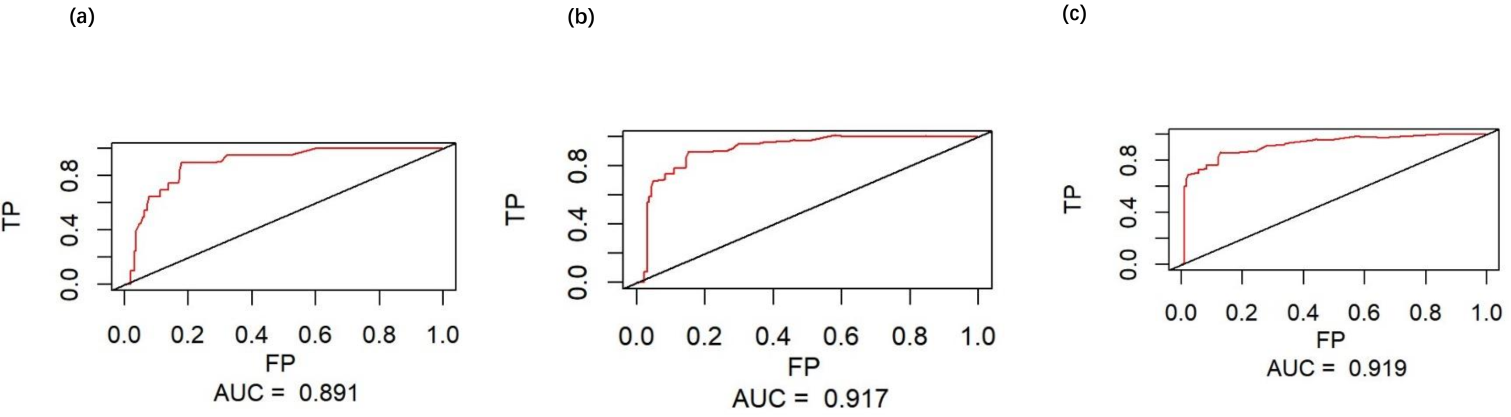

AUC,the area under the curve of the time- dependent receiver operating characteristic curve;PI,prognosis index;TP,true positive FP,flase positive ; KM,Kplan- Meier

Fig 3 ROC(receiver operating characyeristic) curves of internal test set

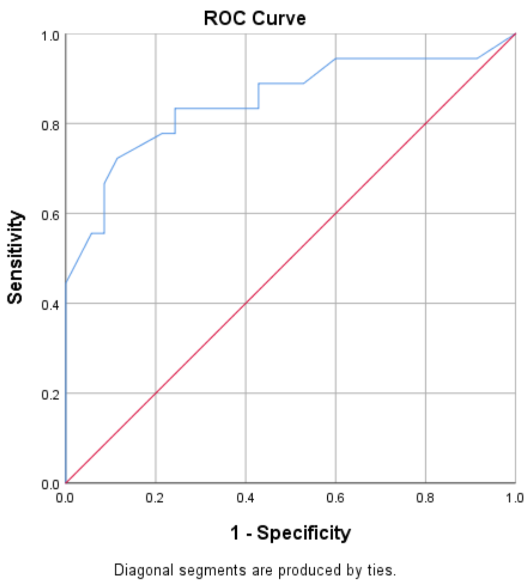

Fig4. Fitted ROC curve of PI index with "KM" method in 1 year (a),2 years(b) and 5 years(c) of internal test set

(a)

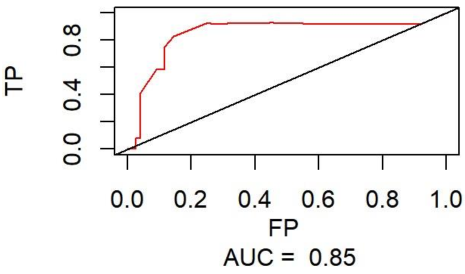

(b)

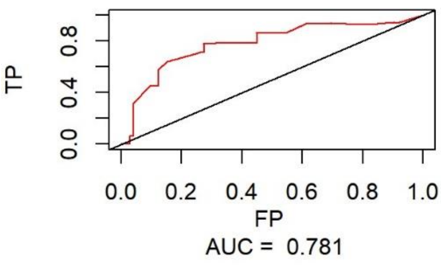

(c)

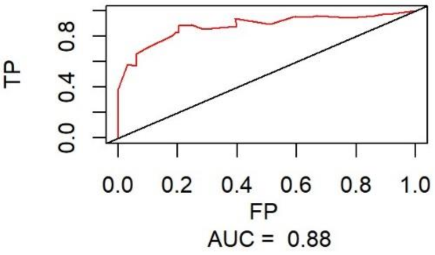

AUC,the area under the curve of the time- dependent receiver operating characteristic curve;PI,prognosis index;TP,true positive FP,flase positive ; KM,Kplan- Meier

Fig 5 ROC curves of external test set

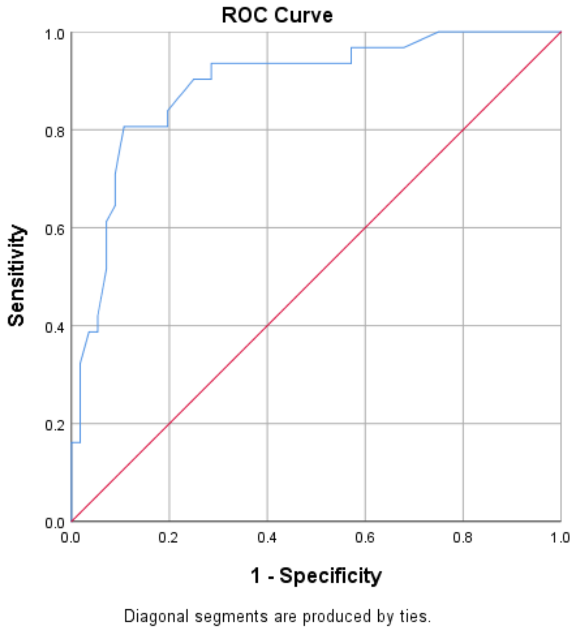

Fig6. Fitted ROC curve of PI index with "KM" method in in 1 year (a),2 years(b) and 5 years(c) of external test set

(a) (b) (c)

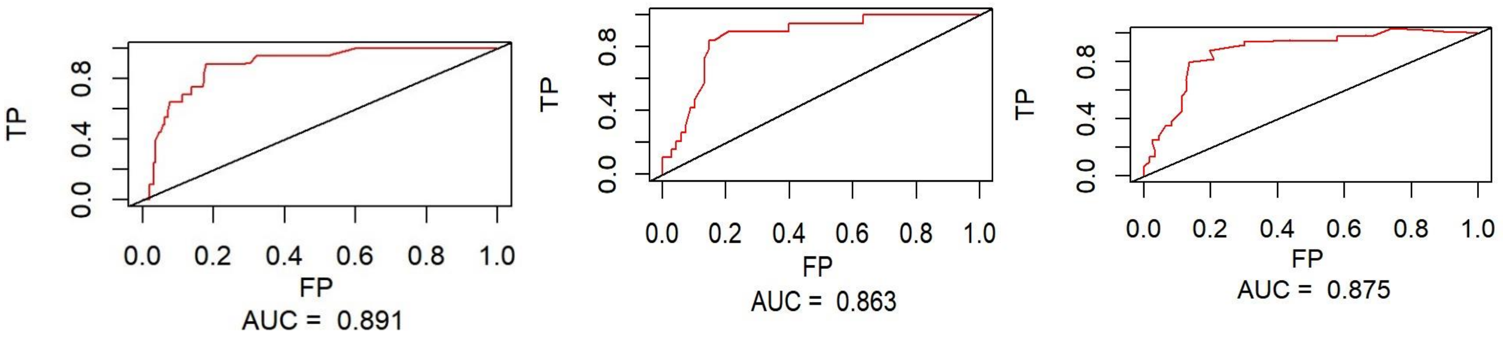

AUC,the area under the curve of the time- dependent receiver operating characteristic curve;PI,prognosis index;TP,true positive FP,flase positive ;KM,Kplan- Meier

Fig 7 Calibration plots for recurrence-freed survival probability at 1(a),2(b),and 5(c) years in internal test set

(a)

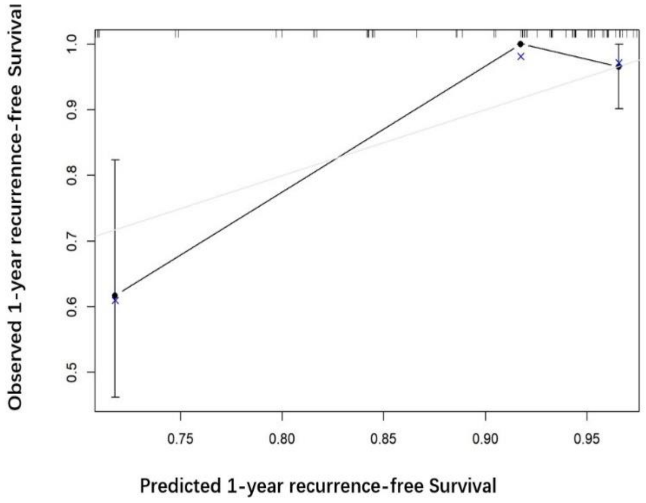

(b)

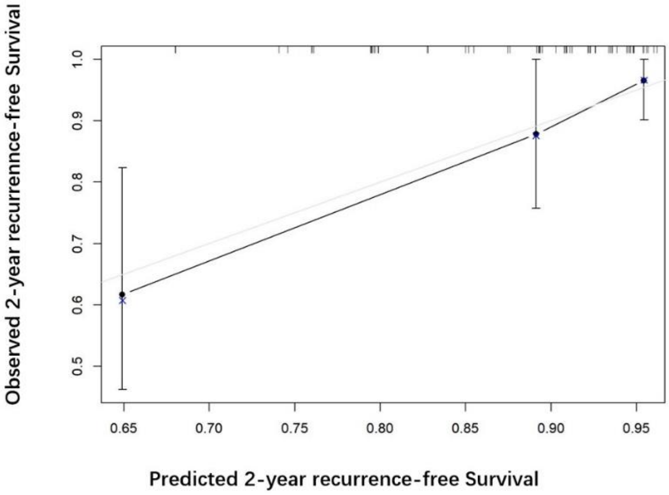

(c)

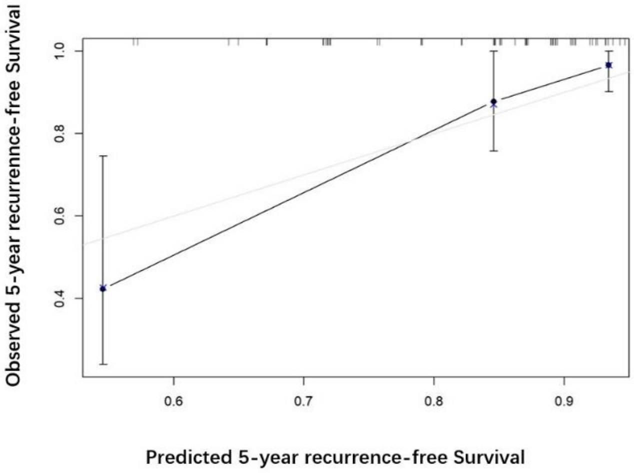

Illustration:The vertical lines represent the 95% confidence intervals of the estimates. The grey lines represents that the ideal lines. Black dot: predicted probabilities according to the nomogram; blue cross: bootstrap- corrected estimates. B=500 repetitions for bootstrapping. RFS, recurrence- free survival

Fig 8 Calibration plots for recurrence-freed survival probability at 1(a),2(b),and 5(c) years in external test set

(a)

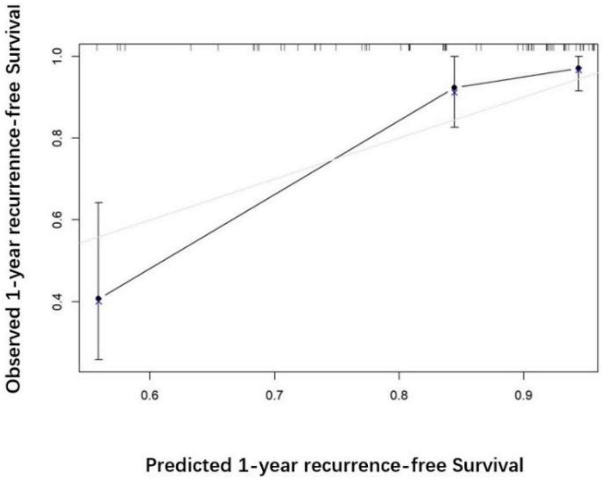

(b)

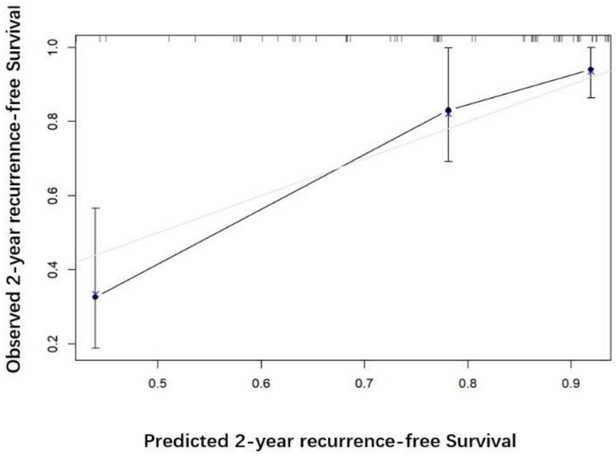

(c)

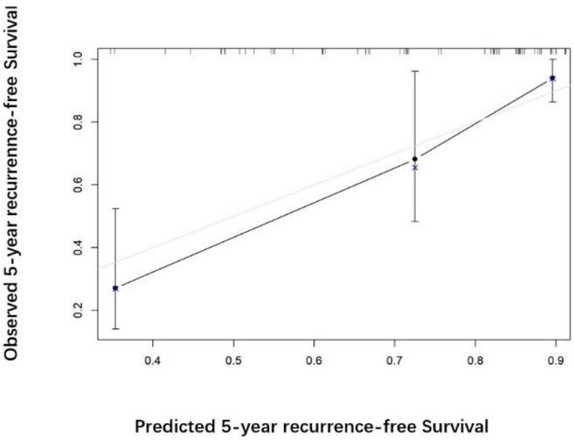

Illustration:The vertical lines represent the 95% confidence intervals of the estimates. The grey lines represents that the ideal lines. Black dot: predicted probabilities according to the nomogram; blue cross: bootstrap- corrected estimates. B=500 repetitions for bootstrapping. RFS ,recurrence- free survival
